# Prevalence of comorbid depression and obesity in general practice: a cross-sectional survey

**DOI:** 10.3399/bjgp14X677482

**Published:** 2014-02-24

**Authors:** Mariko Carey, Hannah Small, Sze Lin Yoong, Allison Boyes, Alessandra Bisquera, Rob Sanson-Fisher

**Affiliations:** Priority Research Centre for Health Behaviour, School of Medicine and Public Health;; Priority Research Centre for Health Behaviour, School of Medicine and Public Health;; School of Medicine and Public Health, University of Newcastle, c/o Hunter New England Population Health, Wallsend, New South Wales, Australia.; Priority Research Centre for Health Behaviour, School of Medicine and Public Health;; Clinical Research Design, IT and Statistical Support, University of Newcastle and Hunter Medical Research Institute, Callaghan, New South Wales, Australia.; Priority Research Centre for Health Behaviour, School of Medicine and Public Health;

**Keywords:** body weight, depression, general practice, obesity, primary care

## Abstract

**Background:**

General practice is a common setting for the provision of weight-management advice, as well as the treatment of depression. While there is some evidence of a reciprocal relationship between obesity and depression, there are limited data about the rates of depression among general practice patients who are underweight, normal weight, overweight, and obese.

**Aim:**

To explore the prevalence of depression among underweight, normal weight, overweight, and obese general practice patients.

**Design and setting:**

A cross-sectional survey was conducted in 12 Australian general practices.

**Method:**

Patients aged ≥18 years and older who were presenting for general practice care were eligible to participate. Consenting patients completed a touchscreen computer survey assessing self-reported weight and height. Depression was assessed by the Patient Health Questionnaire-9 (PHQ-9), with a score of ≥10 used to indicate possible depression.

**Results:**

Data were obtained from 3361 participants. The prevalence of depression was 24% (95% confidence interval [CI] = 11.86 to 39.28) among underweight participants, 11% (95% CI = 8.5 to 14.0) among normal weight participants, 12% (95% CI = 0.9 to 15.2) among overweight participants, and 23% (95% CI = 17.8 to 29.0) among obese participants. The prevalence of depression was higher for women than for men across all weight categories except underweight.

**Conclusion:**

Weight and depression demonstrated a U-shaped relationship, with higher prevalence of depression observed among underweight and obese general practice patients. These conditions may act as red flags for opportunistic screening of depression in the general practice setting.

## INTRODUCTION

Depression and obesity are common, with an estimated 350 million and 500 million people globally with these respective conditions.^1^,^2^ Studies have consistently found a modest association between depression and obesity; however, the causal relationship is unclear. A meta-analysis of 17 community-based studies found that obese people were 1.18 times more likely to have depressive symptoms than those who were not obese, with this association more clearly present among women than men.^3^ Evidence of a reciprocal relationship between depression and excess weight was found in a meta-analysis of longitudinal studies.^4^ Overweight and obesity increased the odds of subsequent depression by 1.27 and 1.55 respectively, while depression increased the odds of obesity at follow-up by 1.58.^4^ This relationship has been suggested to be dose dependent, with a higher body mass index (BMI) being linked to a greater likelihood of clinical depression.^4^,^5^

### Impact of comorbid depression and obesity

Depression and obesity are both associated with social stigma, low self-esteem, and chronic health conditions.^6^–^9^ When depression and obesity co-occur, the adverse health and social consequences are significant. Symptoms of depression in obese patients are strongly associated with poor quality of life, particularly social functioning, emotional roles, and mental health.^10^ Further, obesity coupled with depression has significant economic implications, owing to high service use^11^–^13^ and reduced participation in the labour force.^14^

### GPs’ role in the detection and management of obesity and depression

In many countries, including Australia, the UK, and Canada, clinical practice guidelines recommend that GPs play a role in the management of both obesity and depression.^15^,^16^ Recent data from Australia’s Bettering the Evaluation and Care of Health (BEACH) study of GP encounters found that depression was the fifth most frequently managed problem, and weight and nutrition counselling was the most common preventative treatment administered.^17^ The frequency with which these conditions are encountered in primary care suggests that GPs may need to be equipped to recognise and provide comprehensive care to patients with comorbid depression and obesity.

### Limited information of the prevalence of comorbid depression and obesity

While there are numerous community studies on depression and obesity,^3^,^4^ there are limited data on the prevalence of comorbid obesity and depression in the general practice setting. The frequency with which comorbid depression and obesity occur in general practice is important because of the implications for its detection and management. For example, some somatic criteria for the diagnosis of depression, such as sleep problems, fatigue, or changes in appetite, could be confounded with manifestations of obesity.^6^ While interventions for weight management, such as exercise, may also be helpful in managing depression, there are no protocols for providing integrated treatment of these health conditions. Therefore, if comorbid depression and obesity is a common presentation, the development and testing of models of care to facilitate GPs to recognise and treat these multiple conditions is likely to be indicated. This study aimed to examine the prevalence of depression among underweight, normal weight, overweight, and obese patients in general practice.

**How this fits in**

Previous research suggests a reciprocal relationship between depression and obesity; however, there has been limited study of the relationship between weight and depression in the Australian general practice setting. The results of this study indicate a U-shaped relationship exists between depression and weight, with a higher prevalence of depression observed among both underweight and obese patients. The findings suggest that these conditions may act as red flags for opportunistic screening of depression in the general practice setting.

## METHOD

### Design and setting

A cross-sectional survey was conducted among primary care patients in 12 practices in Australia. Sampling of practices has been described elsewhere.^18^

### Participants

Patients presenting for a primary care appointment, aged ≥18 years, and who had sufficient English to complete the survey independently, were eligible to participate. Those presenting for a nursing or allied health appointment, or who were unable to provide independent informed consent were excluded.

### Procedure

A research assistant sought informed consent from patients to participate in the study when they presented to the practice reception for their appointment. The sex of non-consenters was recorded, to assess consent bias. Consenting patients completed a 15-minute touchscreen computer survey while waiting for their appointment. Patients called into their appointment were able to exit the survey. The survey assessed a range of health risk factors and behaviours; however, only results relating to depression and obesity are presented here. Feedback on health risk factors identified within the survey was not provided to GPs.

### Measures

#### Sociodemographic characteristics

Self-report data were obtained for age category (18–24 years, then in 5-year age categories to ≥70 years), sex, postcode, education, health insurance, and possession of a government Health Care Card or Veterans’ Affairs Card.

#### Medical history

Participants were asked to indicate whether they had ever been told by a doctor or nurse that they had depression. The number of times the participant consulted their GP in the last 12 months was also sought.

#### Body mass index

Participants were asked to report their weight to the nearest kilogram and their height to the nearest centimetre. BMI was calculated and World Health Organization (WHO) definitions applied to categorise underweight (<18.5 kg/m^2^), normal weight (18.5–24.9 kg/m^2^), overweight (25–29.9 kg/m^2^), and obese (>30 kg/m^2^).^19^ BMI derived from self-reported weight and height has been shown to have adequate reliability when compared to BMI derived from measured values.^20^

#### Depression

The Patient Health Questionnaire (PHQ-9) was used to assess depression. This brief tool has been used extensively in the primary care setting.^21^ It is scored on a 27-point scale.^22^ A total score of ≥10 to classify depression is considered to provide the best balance between sensitivity (88%) and specificity (88%) compared to mental health professional assessment, and this cut-off value was used for this study.^22^

### Data analyses

The proportions and modified Clopper–Pearson (exact) 95% confidence intervals (CIs, to account for clustering by practice) were calculated for each BMI category (underweight, normal weight, overweight, obese) for both depressed and non-depressed patients. The prevalence of depression across BMI categories was also calculated separately for men and women. For those who were identified as depressed, a χ^2^ test was undertaken, to compare the proportion who consulted their GP three or more times and the proportion reporting a previous history of depression, by BMI category. A Rao–Scott χ^2^ test was used to test whether the distribution of depressed patients was equal across all BMI categories.

## RESULTS

### Practice consent rate and characteristics

Of 48 practices approached to participate in the study, 12 (25%) consented. Eight practices had a practice nurse. The mean number of GPs employed within participating practices was 5 (range 3 to 15).

### Patient consent rate and characteristics

Of 5667 patients screened for eligibility, 4705 were eligible and, of these, 4058 (86%) consented to participate. Comparison of the percentage of males among non-consenting patients with that among consenting patients found no significant differences between the two groups (χ^2^ = 0.13; degrees of freedom [df] = 1; *P* = 0.253). Participants with self-reported height greater than 240 cm and less than 120 cm, and weight greater than 250 kg and less than 30 kg were excluded from the analyses, as these values were perceived to be errors in self-report. This left a total of 3361 participants. As shown in Table 1, the majority of participants were female (61%) and had attended the practice on a previous occasion (94%). Twenty-two per cent were aged 70 years or older, and 25% reported one or more chronic medical conditions.

**Table 1:** Demographic and medical characteristics of participants (*n* = 3361)

**Characteristic**	***n* (%)**
Sex (*n* = 3361)	
Male	1305 (39)

Age, years (*n* = 3361)	
18–29	439 (13)
30–39	464 (14)
40–49	570 (17)
50–59	587 (17)
60–69	577 (17)
≥70	724 (22)

Education (*n* = 3120)^a^	
Primary	49 (1.6)
Some high school	239 (7.7)
Year 10	456 (15)
Completed high school	533 (17)
Technical and further education/diploma	535 (17)
University	1003 (32)
Postgraduate	220 (7.1)
Other	85 (2.7)

Visited clinic before? (*n* = 3151)^a^	
Yes	2969 (94)

Veterans’ Affairs Card (*n* = 3361)	86 (2.6)

Health Care Card (*n* = 3361)	742 (22)

Private healthcare insurance (*n* = 3361)	1953 (58)

Number of chronic conditions (*n* = 3361)	
None	2534 (75)
1–2	792 (24)
≥3	35 (1.0)

a Number less than total participants, owing to missing data.

### Prevalence of depression among underweight, normal weight, overweight, and obese participants

As shown in Table 2, the distribution of the prevalence of depression by weight category was U-shaped, with the highest rates observed among underweight and obese participants (24% and 23% respectively). The prevalence of depression was also remarkably similar among those of normal weight and those who were overweight (11% and 12% respectively). The rates of depression varied significantly by weight category (χ^2^ = 109.5; df = 3; *P*<0.0001).

**Table 2:** Percentage of patients classified as depressed (PHQ-9≥10) by weight category

**Weight category**	**Not depressed (PHQ-9<10) *n* (%) [95% CI]**	**Depressed (PHQ-9 ≥10) *n* (%) [95% CI]**	**Total**
Underweight	68 (76) [60.72 to 88.14]	21 (24) [11.86 to 39.28]	89
Normal weight	1265 (89) [86.0 to 91.5]	157 (11) [8.5 to 14.0]	1422
Overweight	1035 (88) [84.8 to 91.1]	138 (12) [8.9 to 15.2]	1173
Obese	521 (77) [71.0 to 82.2]	156 (23) [17.8 to 29.0]	677

PHQ-9 = Patient Health Questionnaire.

### Depression among males and females, by BMI category

**Figure 1.**
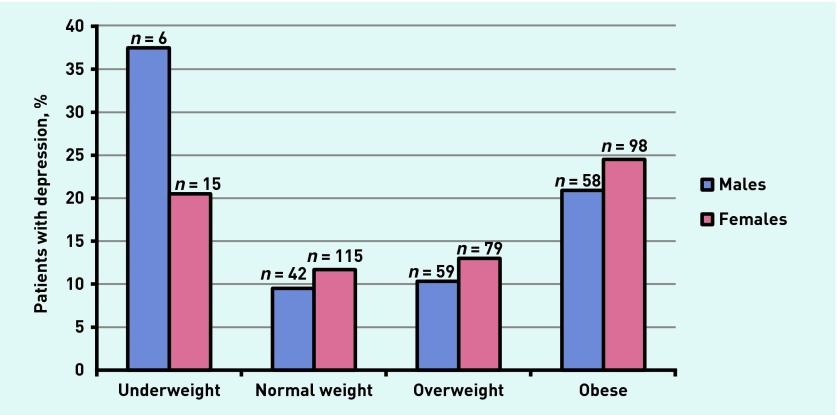
***Percentage of males and females with depression, by BMI category.***

As shown in Figure 1, there was a higher percentage of females than males with depression in all BMI categories except for the underweight category. However, the prevalence of underweight among males was very low (*n* = 6, 1.2% of males).

### Consulting behaviour and history of depression among people with depression, by BMI category

The number and proportion of patients with depression who reported a history of depression was calculated for the categories underweight (*n* = 10, 48%), normal weight (*n* = 92, 59%), overweight (*n* = 79, 57%), and obese (*n* = 109, 70%). There were no significant differences by weight category (χ^2^ = 6.97; df = 3; *P* = 0.0729). Similarly, there were no significant differences across weight categories for the proportion of depressed patients who reported three or more GP consultations within the past 12 months (χ^2^ = 2.02; df = 3; *P* = 0.5679). The number and proportion of responders with depression who had three more consultations within the past 12 months were: underweight (*n* = 13, 62%), normal weight (*n* = 108, 69%), overweight (*n* = 98, 71%), and obese (*n* = 112, 72%).

## DISCUSSION

### Summary

To the authors’ knowledge, this is one of the first studies to examine the prevalence of depression across all BMI categories in the Australian general practice setting. Depression and weight demonstrated a U-shaped relationship, with rates of depression higher among those who were underweight and among those who were obese. Depression was more prevalent among women across all BMI categories except underweight.

### Strengths and limitations

The multi-site nature of the current study is a strength; however, the low practice consent rate raises the possibility that participating practices were not representative of the general practice setting. This is mitigated by a high participant consent rate, indicating that the sample was representative of participating practices.

The use of self-reported data on height and weight to calculate BMI may have resulted in an underestimation of BMI. However, the reliability of self-reported weight and height data collected for the current study were assessed with measured data collected for a subset of participants, and substantial agreement was found between self-report and measured categorisation of BMI.^20^ Given the low number of people reporting both underweight and depression in the current study, the results pertaining to this group must be treated with caution. The PHQ-9 has been shown to have high sensitivity and specificity in the general practice setting. However, it is a screening tool, and does not provide a clinical diagnosis of depression. Therefore, there is likely to be some degree of measurement error in the rates of depression reported for each BMI category.

### Comparison with existing literature

While there are limited studies in the general practice setting, two large community-based studies conducted in the US and the Netherlands, involving 40 086 and 43 534 participants respectively, reported similar U-shaped associations between BMI and depression.^23^,^24^ Consistent with the current study, both studies used self-reported measures to collect data on height, weight, and depressive symptoms.

The point prevalence of depression among obese patients in the current study was 23% and is higher than the 14% reported in a previous study from one family practice clinic in the US.^25^ In the latter study, depression rates were based on a documented diagnosis of depression in the electronic medical record. The strong evidence that depression is under-detected in primary care^26^ is likely to account for the difference in prevalence reported. Past research sheds some light on the possible reasons for the high prevalence of depression among obese people. Factors associated with obesity, such as stigmatisation; discrimination in health care, education, and employment;^27^,^28^ low self-esteem; and body dissatisfaction,^4^,^6^ can contribute to or exacerbate depressive illness in an obese person.^4^ Conversely, features of depression may also cause or exacerbate weight problems and obesity. This includes prominent symptoms of depression, such as physical inactivity and weight gain, as well as weight gain attributed to antidepressant medication, major depressive episodes, or endocrine disturbance.^4^,^8^,^29^

Previous studies that have assessed the association between underweight and depression have focused specifically on individuals with eating disorders, which may not accurately reflect the general population of underweight individuals.^30^ Therefore, there are limited data with which to compare the present finding of a 24% prevalence rate for depression among underweight individuals. The few community studies that have examined the association between depression and underweight report inconsistent results.^24^,^30^ Depressive disorders are characterised by changes in eating behaviour, both overeating and under-eating, and weight changes, both significant weight gain and loss, and this is listed as a core diagnostic criterion according to the *Diagnostic and statistical manual of mental disorders* (DSM-5).^31^ While a decrease in food intake might be less common than an increase in food intake, these behaviours could partially explain the U-shaped association between depression and body weight.^24^

Rates of depression were higher among women than men across all BMI categories, except for the underweight category. However, it should be noted that only a small number of men were underweight (*n* = 6, 1.2%); therefore, these data must be interpreted with caution. An association between psychopathology and underweight among men has also been reported in community samples,^23^ suggesting that the current finding warrants further investigation.

### Implications for research and practice

Given the paucity of prior research examining the prevalence of depression by BMI categories among general practice attendees, further research is needed to confirm the current findings and to determine the extent to which they can be generalised within the primary care setting in Australia and overseas. The data from this study indicated that rates of depression history among people with elevated PHQ-9 scores were similar across BMI categories. Similarly, there were no differences in the rate of depressed people who consulted their GP on three or more occasions in the past 12 months. Therefore, it would be useful for future research to investigate whether other factors may discriminate between those who are underweight and depressed and those who are obese and depressed. This may provide some insights into the impact of depression when associated with underweight or obesity, and assist in developing intervention approaches in this setting.

Both underweight and obesity may act a potential red flag for opportunistic screening for depression. The relatively high rates of depression among both underweight and obese participants in the present study suggest that these conditions may need to be addressed in combination. Given that both conditions are associated with stigma,^27^,^28^,^32^ GPs may be reluctant to raise issues of weight and/or depression with patients for whom this is not the primary reason for presenting for care.^33^ Therefore, exploration into the most effective ways of discussing the potential comorbidity of obesity and depression is needed.

In addition to aiding weight management,^34^ there is some evidence suggesting that exercise may improve mood.^35^ Therefore, exercise interventions integrated into depression treatment may be valuable among depressed patients who are underweight or obese. Similarly, depression screening and treatment may be beneficial alongside health-behaviour interventions for obesity. Despite their co-occurrence, there are no models for providing combined treatment for depression and obesity, and this is an important area for research. One of the key questions that remain unanswered is whether it is more effective to target these conditions simultaneously or sequentially. The importance of developing evidence-based approaches for tackling these two major health problems in an integrated way is highlighted by the present finding that almost one-quarter of those who were obese or underweight reported depression.
